# Crystal structure of a putative short-chain dehydrogenase/reductase from *Paraburkholderia xenovorans*

**DOI:** 10.1107/S2053230X21012632

**Published:** 2022-01-01

**Authors:** Jaysón Davidson, Kyndall Nicholas, Jeremy Young, Deborah G. Conrady, Stephen Mayclin, Sandhya Subramanian, Bart L. Staker, Peter J. Myler, Oluwatoyin A. Asojo

**Affiliations:** a Department of Chemistry and Biochemistry, Hampton University, 200 William R. Harvey Way, Hampton, VA 23668, USA; b UCB Pharma, Bedford, Massachusetts, USA; c Seattle Structural Genomics Center for Infectious Disease (SSGCID), Seattle, Washington, USA; d Center for Global Infectious Disease Research, Seattle Children’s Research Institute, 307 Westlake Avenue North Suite 500, Seattle, WA 98109, USA

**Keywords:** SSGCID, structural genomics, *Paraburkholderia xenovorans*, oxidoreductases, education and training, detoxification, Seattle Structural Genomics Center for Infectious Disease

## Abstract

*Paraburkholderia xenovorans* degrades organic wastes, including polychlorin­ated biphenyls. The atomic structure of a putative dehydrogenase/reductase (SDR) from *P. xenovorans* (*Px*SDR) was determined in space group *P*2_1_ at a resolution of 1.45 Å. *Px*SDR shares less than 37% sequence identity with any known structure and assembles as a prototypical SDR tetramer. As expected, there is some conformational flexibility and difference in the substrate-binding cavity, which explains the substrate specificity. Uniquely, the cofactor-binding cavity of *Px*SDR is not well conserved and differs from those of other SDRs. *Px*SDR has an additional seven amino acids that form an additional unique loop within the cofactor-binding cavity. Further studies are required to determine how these differences affect the enzymatic functions of the SDR.

## Abstract 2

The high-resolution structure of a putative short-chain reductase from the commercially important bacterium *Paraburkholderia xenovorans* is reported. *P. xenovorans* degrades organic wastes such as polychlorinated biphenyls.

## 1. Introduction

*Paraburkholderia xenovorans* is a model bacterium used to study unique pathways, notably the ability of this genus of bacteria to degrade polychlorinated biphenyls and other organic waste products (Francova *et al.*, 2004 ▸; Tehrani *et al.*, 2014 ▸). The genome of *P. xenovorans* has been sequenced and is one of the largest bacterial genomes studied to date. *P. xenovorans* has diverse functions, including nitrogen fix­ation, the catabolysis of aromatic compounds and the degradation of various organic wastes (Francova *et al.*, 2004 ▸; Tehrani *et al.*, 2014 ▸). However, the underlying mechanisms of many of these processes are poorly understood. Additionally, unlike some other *Paraburkholderia* species, *P. xenovorans* is not pathogenic to humans. The Seattle Structural Genomics Center for Infectious Disease (SSGCID) selected targets from *P. xenovorans* for high-throughput structural studies to increase the breadth of drug-target structures to aid drug development against human pathogenic *Burkholderia* species such as *B. mallei* and *B. pseudomallei*. Here, we present the structure of one of these target proteins, a short-chain de­hydrogenase/reductase (SDR) which shares less than 37% sequence identity with any published structure. *P. xenovorans* SDR (*Px*SDR) is predicted to be a type II fatty-acid synthetase and NAD(P)(H)-dependent oxidoreductase involved in the metabolism of diverse molecules, including lipids, amino acids, carbohydrates, cofactors, hormones and xenobiotics, or other compounds. The structure reported here may offer insights into the metabolism of small molecules by *P. xenovorans.*

## 2. Materials and methods

### 2.1. Macromolecule production

Cloning, expression and purification were conducted as part of the Seattle Structural Genomics Center for Infectious Disease (SSGCID; Myler *et al.*, 2009 ▸; Stacy *et al.*, 2011 ▸) following standard protocols described previously (Bryan *et al.*, 2011 ▸; Choi *et al.*, 2011 ▸; Serbzhinskiy *et al.*, 2015 ▸). The full-length putative short-chain reductase (*Px*SDR, UniProt Q13GE3) was PCR-amplified from genomic DNA using the primers shown in Table 1 ▸. The resultant amplicon was cloned into the ligation-independent cloning (LIC; Aslanidis & de Jong, 1990 ▸) expression vector pBG1861 encoding a non­cleavable 6×His fusion tag (MAHHHHHHM-ORF). Plasmid DNA was transformed into chemically competent *Escherichia coli* BL21(DE3)R3 Rosetta cells. The plasmid containing His-*Px*SDR was expression-tested and 2 l of culture was grown using auto-induction medium (Studier, 2005 ▸) in a LEX Bio­reactor (Epiphyte Three Inc.). The expression clone for *Px*SDR, BuxeA.00010.c.B1.GE39410, is available at https://www.ssgcid.org/available-materials/expression-clones/.

His-*Px*SDR was purified in a two-step protocol consisting of an immobilized metal-affinity chromatography (IMAC) step and size-exclusion chromatography (SEC). All chromatography runs were performed on an ÄKTApurifier 10 (GE) using automated IMAC and SEC programs according to previously described procedures (Bryan *et al.*, 2011 ▸). Thawed bacterial pellets were lysed by sonication in 200 ml buffer consisting of 25 m*M* HEPES pH 7.0, 500 m*M* NaCl, 5% glycerol, 0.5% CHAPS, 30 m*M* imidazole, 10 m*M* MgCl_2_, 1 m*M* TCEP, 250 µg ml^−1^ AEBSF, 0.025% azide. After sonication, the crude lysate was clarified with 20 µl (25 units µl^−1^) benzonase and incubated while mixing at room temperature for 45 min. The lysate was then clarified by centrifugation at 10 000 rev min^−1^ for 1 h using a Sorvall centrifuge (Thermo Scientific). The clarified supernatant was then passed over an Ni–NTA HisTrap FF 5 ml column (GE Healthcare) which was pre-equilibrated with loading buffer composed of 25 m*M* HEPES pH 7.0, 500 m*M* NaCl, 5% glycerol, 30 m*M* imidazole, 1 m*M* TCEP, 0.025% sodium azide. The column was washed with 20 column volumes (CV) of loading buffer and was eluted with loading buffer plus 250 m*M* imidazole in a linear gradient over 7 CV. Peak fractions, as determined by UV absorption at 280 nm, were pooled and concentrated to 5 ml. A SEC column (Superdex 75, GE) was equilibrated with running buffer composed of 25 m*M* HEPES pH 7.0, 500 m*M* NaCl, 5% glycerol, 2 m*M* DTT, 0.025% azide. The peak fractions were collected and analyzed for the protein of interest using SDS–PAGE. The SEC peak fractions eluted as a single large peak at a molecular mass of ∼76 kDa, suggesting a dimeric enzyme. The peak fractions were pooled and concentrated to 45 mg ml^−1^ using an Amicon purification system (Millipore). Aliquots of 200 µl were flash-frozen in liquid nitrogen and stored at −193 K until use for crystallization.

### 2.2. Crystallization

*Px*SDR was crystallized by the sitting-drop vapor-diffusion method using the JCSG+ commercial crystallization screen (Rigaku Reagents). Crystals were obtained by mixing 0.4 µl protein solution at 22.5 mg ml^−1^ with 0.4 µl precipitant (Rigaku Reagents JCSG+ screen condition H11: 0.2 *M* magnesium chloride, 0.1 *M* bis-Tris pH 5.5, 25% PEG 3350), equilibrating against a reservoir consisting of 80 µl precipitant and incubating at 287 K (Table 2 ▸). A single crystal was transferred into a cryoprotectant (reservoir solution supplemented with 20% ethylene glycol) and vitrified by plunging into liquid nitrogen before data collection.

### 2.3. Data collection and processing

X-ray diffraction data were collected on LS-CAT beamline 21-ID-F at the Advanced Photon Source. Data were integrated using *XDS* and reduced with *XSCALE* (Kabash, 2010 ▸). Additional data-collection information is provided in Table 3 ▸. The raw images and detailed data-collection information are available for download (https://proteindiffraction.org/project/5jc8/).

### 2.4. Structure solution and refinement

The structure of *Px*SDR was solved by molecular replacement with *MOLREP* (Vagin & Teplyakov, 2010 ▸; Lebedev *et al.*, 2008 ▸) using PDB entry 4ni5 (36% sequence identity), an unpublished structure from the SSGCID, as a search model. The structure was refined in *Phenix* (Liebschner *et al.*, 2010 ▸) with manual model building in *Coot* (Emsley & Cowtan, 2004 ▸; Emsley *et al.*, 2010 ▸). The quality of the model was assessed using *MolProbity* (Headd *et al.*, 2009 ▸) and structure-refinement statistics are provided in Table 4 ▸. The structure was deposited in the Protein Data Bank as entry 5jc8. All structure figures were made using *PyMOL* (DeLano, 2002 ▸).

## 3. Results and discussion

**Figure 1:**
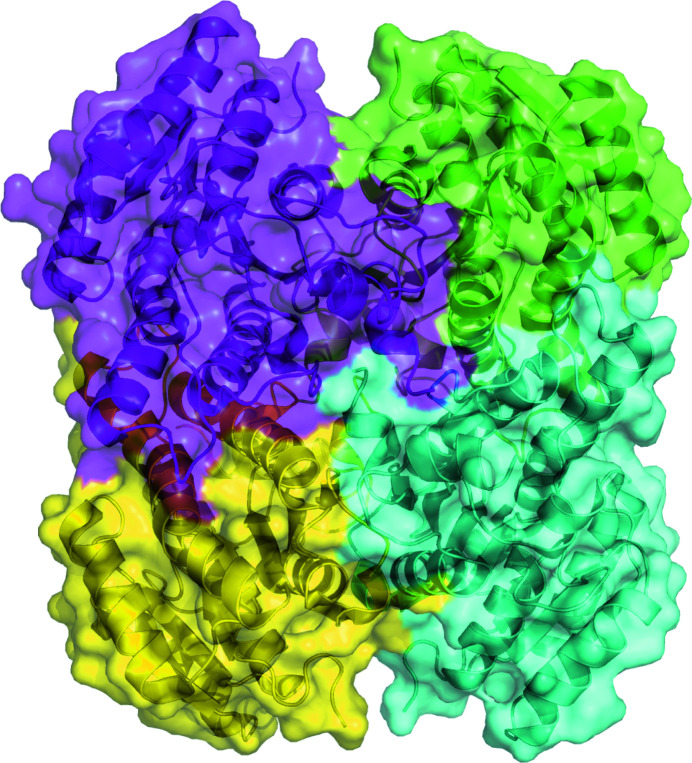
*Px*SDR is a prototypyical SDR tetramer.

**Figure 2:**
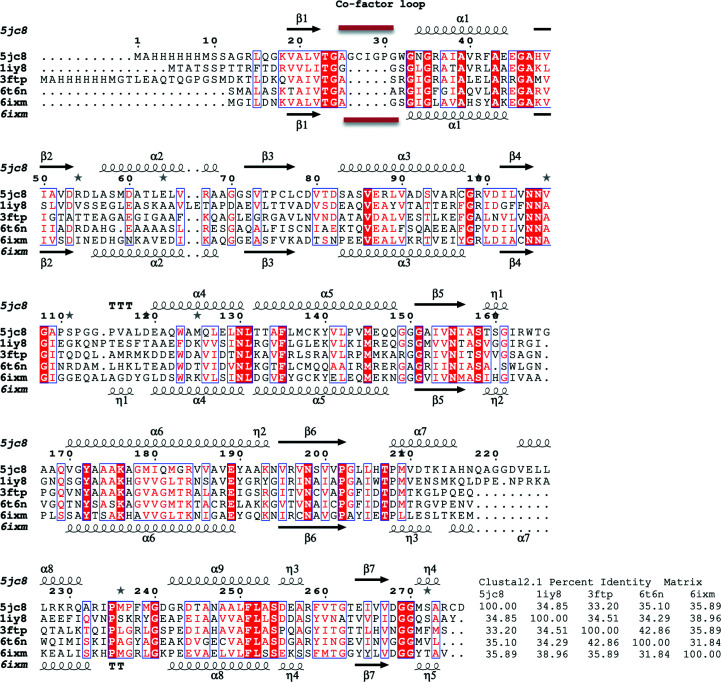
Structural and primary-sequence alignment of *Px*SDR with the closest structures identified by *PDBeFold*. Also shown is the percent identity matrix generated with *Clustal*2.1. The structures are PDB entry 5jc8 (the apo structure of *Px*SDR), PDB entry 1iy8 (the crystal structure of levodione reductase from *Leifsonia aquatica*), PDB entry 3ftp [3-ketoacyl-(acyl-carrier-protein) reductase from *Burkholderia pseudomallei*], PDB entry 6t6n [*Klebsiella pneumoniae* FabG2(NADH-dependent) in complex with NADH] and PDB entry 6ixm (ketone reductase ChKRED20 from the genome of *Chryseobacterium*). The secondary-structure elements shown are α-helices (α), 3_10_-helices (η), β-strands (β) and β-turns (TT). Identical residues are shown in white on a red background and conserved residues are shown in red. This figure was generated using *ESPript* (Gouet *et al.*, 1999 ▸, 2003 ▸).

The reported apo structure of *Px*SDR was determined in the monoclinic space group *P*2_1_ as a prototypical SDR tetramer (Fig. 1 ▸). Each monomer has the prototypical NADPH Rossmann topology as observed in the architecture of PFAM domain PF00106 or the short-chain dehydrogenases. Specifically, *Px*SDR has the enoyl-(acyl carrier protein) reductase or 3-oxoacyl-ACP reductase domain architecture otherwise referred to as adh_short_C2. The most similar structures to *Px*SDR were identified by *PDBeFold* (http://www.ebi.ac.uk/msd-srv/ssm) analysis using the default threshold cutoffs of 70% for the percentage of secondary structure of the target chain identified in the query protein and of the secondary structure of the query chain (Krissinel & Henrick, 2004 ▸). The closest structure is that of the ketone reductase ChKRED20 from the genome of *Chryseobacterium* (Li *et al.*, 2019 ▸). This enzyme shares a sequence identity of <36%, with an r.m.s.d. of 1.24 Å for 89% of the matched sequence identity. Wild-type ChKRED20 is an NADH-dependent ketoreductase that reduces over 100 g l^−1^ ketones for some pharmaceutically relevant substrates and can use 2-propanol as the ultimate reducing agent. All of the closest structures have less than 36% sequence similarity to *Px*SDR (Fig. 2 ▸). These structures are PDB entry 1iy8, the crystal structure of levodione reductase from *Leifsonia aquatica* (Sogabe *et al.*, 2003 ▸), PDB entry 3ftp, a 3-ketoacyl-(acyl-carrier-protein) reductase from *Burkholderia pseudomallei* (Baugh *et al.*, 2013 ▸), PDB entry 6t6n, *Klebsiella pneumoniae* FabG2(NADH-dependent) in complex with NADH (Vella *et al.*, 2021 ▸), and PDB entry 6ixm, ketone reductase ChKRED20 from the genome of *Chryseobacterium* (Li *et al.*, 2019 ▸). Interestingly, while all of the other structures have well conserved cofactor-binding domains, an extended loop connecting the first strand in the N-terminus to the first helix creates a larger cofactor-binding cavity in *Px*SDR (Figs. 2 ▸ and 3 ▸). This additional structural difference between *Px*SDR and the other structures in the cofactor-binding domain is unique. This is an unexpected difference between *Px*SDR and the other proteins beyond the expected flexibility in proximity to the substrate-binding cavity. While the flexibility in the substrate-binding cavity explains the specificity of each protein, it is unknown why *Px*SDR has this unique insertion in the cofactor loop (Figs. 2 ▸ and 3 ▸).

## 4. Conclusions

While having a prototypical SDR topology, the apo structure of *Px*SDR reveals conformational flexibility in both the cofactor- and substrate-binding cavities that needs to be further investigated in order to determine the roles of this enzyme in the degradation of organic wastes by *P. xenovorans*.

## Supplementary Material

PDB reference: putative short-chain reductase, 5jc8

## Figures and Tables

**Figure 3:**
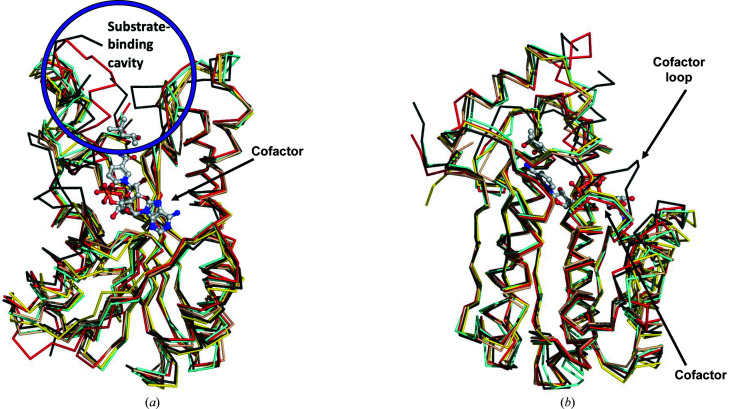
*Px*SDR superposed on its closest structural orthologues reveals differences in the substrate- and cofactor-binding cavities. (*a*) The substrate-binding cavities of the proteins, indicated in the blue circle, have differences that are indicative of different substrate specificities. (*b*) *Px*SDR in black has a unique loop insertion in the cofactor-binding cavity, while the other proteins have a well conserved cofactor-binding cavity and loops. The superposed structures are PDB entry 5jc8 (apo structure of *Px*SDR, black), PDB entry 1iy8 (crystal structure of levodione reductase from *Leifsonia aquatica*, red), PDB entry 3ftp [3-ketoacyl-(acyl-carrier-protein) reductase from *Burkholderia pseudomallei*, yellow], PDB entry 6t6n [*Klebsiella pneumoniae* FabG2(NADH-dependent) in complex with NADH, wheat] and PDB entry 6ixm (ketone reductase ChKRED20 from the genome of *Chryseobacterium*, aquamarine). The cofactor, NADH and substrate, (4*R*)-2-methylpentane-2,4-diol, are from PDB entry 1iy8. Structures were superposed with *PyMOL*.

**Table 1:** Macromolecule-production information

Source organism	*Paraburkholderia xenovorans* (strain LB400)
DNA source	Genomic DNA, provided by Dr Mary Lidstrom (University of Washington, USA)
Forward primer	5′-CTCACCACCACCACCACCATATGAGTTCAGCAGGAAGATTGCAG-3′
Reverse primer	5′-ATCCTATCTTACTCACTTAATCGCAGCGGGCGCTCATCC-3′
Expression vector	pBG1861
Expression host	*Escherichia coli* BL21(DE3)R3 Rosetta cells
Complete amino-acid sequence of the construct produced	MAHHHHHHMSSAGRLQGKVALVTGAGCIGPGWGNGRAIAVRFAEEGAHVIAVDRDLASMDATLELVRAAGGSVTPCLCDVTDSASVERLVADSVARCGRVDILVNNVGAPSPGGPVALDEAQWAMQLELNLTTAFLMCKYVLPVMEQQGGGAIVNIASTSGIRWTGAAQVGYAAAKAGMIQMGRVVAVEYAAKNVRVNSVVPGLLHTPMVDTKIAHNQAGGDVELLLRKRQARIPMPFMGDGRDTANAALFLASDEARFVTGTEIVVDGGMSARCD

**Table 2:** Crystallization

Method	Vapor diffusion, sitting drop
Plate type	96-well, Compact 300, Rigaku
Temperature (K)	287
Protein concentration	*Px*SDR (BuxeA.00010.c.B1.PS02595) at 22.5 mg ml^−1^
Buffer composition of protein solution	25 m*M* HEPES pH 7.0, 500 m*M* NaCl, 5% glycerol, 2 m*M* DTT, 0.025% azide
Composition of reservoir solution	Rigaku Reagents JCSG+ screen H11: 0.2 *M* magnesium chloride, 0.1 *M* bis-Tris pH 5.5, 25% PEG 3350
Volume and ratio of drop	0.4 µl protein:0.4 µl reservoir (1:1)
Volume of reservoir (µl)	80

**Table 3:** Data collection and processing Values in parentheses are for the outer shell.

Diffraction source	Beamline 21-ID-F, APS
Wavelength (Å)	0.97872
Temperature (K)	100
Detector	RayoniX MX-225 CCD
Crystal-to-detector distance (mm)	140
Rotation range per image (°)	1
Total rotation range (°)	240
Exposure time per image (s)	1
Space group	*P*2_1_
*a*, *b*, *c* (Å)	71.18, 81.08, 86.37
α, β, γ (°)	90, 94.53, 90
Mosaicity (°)	0.145
Resolution range (Å)	50–1.45 (1.49–1.45)
Total No. of reflections	855886 (53861)
No. of unique reflections	165687 (10796)
Completeness (%)	95.8 (84.8)
Multiplicity	5.2 (5.0)
〈*I*/σ(*I*)〉	25.10 (4.45)
*R*_r.i.m._ †	0.042 (0.392)
Overall *B* factor from Wilson plot (Å^2^)	12.76

† Estimated *R*_r.i.m._ = *R*_merge_[*N*/(*N* − 1)]^1/2^, where *N* is the data multiplicity.

**Table 4:** Structure solution and refinement Values in parentheses are for the outer shell

Resolution range (Å)	50–1.45 (1.48–1.45)
Completeness (%)	95.8
σ Cutoff	*F* > 1.34σ(*F*)
No. of reflections, working set	165652 (9476)
No. of reflections, test set	2032 (112)
Final *R*_cryst_	0.141 (0.194)
Final *R*_free_	0.158 (0.202)
No. of non-H atoms	
Protein	7374
Ion	4
Ligand	66
Solvent	1068
Total	8512
R.m.s. deviations	
Bonds (Å)	0.007
Angles (°)	0.878
Average *B* factors (Å^2^)	
Protein	17.5
Ion	17.7
Ligand	33.7
Water	31.7
Ramachandran plot	
Most favored (%)	96
Allowed (%)	4
